# Commentary: Oral fluid testing can be used to monitor xenotransplant donor herds for porcine cytomegalovirus/roseolovirus status

**DOI:** 10.3389/fvets.2025.1571657

**Published:** 2025-05-14

**Authors:** Joachim Denner

**Affiliations:** Institute of Virology, Free University Berlin, Berlin, Germany

**Keywords:** porcine cytomegalovirus/porcine roseolovirus, detection methods, PCR, immunological methods, xenotransplantation

## Introduction

To address the shortage of human tissues and organs for treating organ failure, xenotransplantation is progressing toward clinical application. Pigs have been chosen as donor species for various reasons, and extensive genetic modifications are being implemented to prevent xenotransplant rejection. However, xenotransplantation may be associated with the transmission of pathogenic pig microorganisms. One such pathogen is porcine cytomegalovirus/porcine roseolovirus (PCMV/PRV), which has been shown to significantly reduce the survival time of pig organs in non-human primates (1). Although originally named PCMV because of the morphological similarities between infected cells and those infected with human cytomegalovirus (HCMV), subsequent research revealed that PCMV/PRV is a roseolovirus related to human herpesviruses 6 and 7, not HCMV. The International Committee on Taxonomy of Viruses (ICTV) has officially designated the virus as suid herpesvirus 2 (SuHV-2). This virus was also transmitted to the first human recipient of a genetically modified pig heart and contributed to his death (2). The virus had not been detected in the donor pig of the transplanted heart due to the use of an inappropriate detection method. A nasal swab from this donor pig has been tested using PCR; however, the virus can be detected using nasal swabs only in newly infected animals experiencing rhinitis (3). In one experiment infecting pigs with PCMV/PRV, the maximum duration of nasal virus excretion was recorded at 32 days (4). Therefore, for future clinical xenotransplantations, it is essential to implement highly sensitive and appropriate detection methods.

## Appropriate methods to detect PCMV/PRV

Recently, Schommer et al. (5) investigated the feasibility of using oral fluids and real-time PCR for PCMV/PRV screening. After screening animals from the National Swine Research and Resource Center herd using spleen tissue, blood, and oral fluids, they concluded that oral fluid testing could be used to monitor xenotransplant donor herds for PCMV/PRV. However, a closer analysis of their results and previous publications suggests that this conclusion may not be entirely accurate.

Not all animals in their study that tested positive for PCMV/PRV in spleen tissue were also positive in oral fluid testing in their experiments (Table 1) (5). The spleen tissue was chosen as a reference because, in some studies, it was the pig organ where the virus was most frequently detected (6). However, caution is needed here as well. In other publications, different organs—such as the nose and kidney—were reported to have the highest PCR-based detection of the virus (7). Even when combining oral fluid and blood testing, some animals that tested positive for the spleen remained undetected. This correlates with previously published data that testing adult pigs rarely gave positive results when testing oral swabs (6) (Table 1). The likelihood of detecting PCMV/PRV using this method was higher in juvenile pigs aged 3–5 weeks. A very high prevalence of the virus, detectable by PCR, was observed in 10-day-old piglets, likely infected after birth by their mother sows (8). In adult pigs, the detection rate is significantly lower or zero due to the virus entering a latent stage. In the last two studies, oral swabs were used instead of oral fluids. Oral swabs, along with nasal swabs, are taken from a smaller area, while oral fluids are collected from ropes after the pigs chew on them.

**Table 1:** Comparison of sample types for detecting PCMV/PRV.

**Pigs**	**Samples used for PCMV/PRV detection and percentage of positive animals tested by real-time PCR**						**References**
	**Oral fluid/oral swabs**	**Spleen**	**Blood**	**Serum**	**Ear biopsies**	**Anal swabs**	
3 months to 2-year-old pigs^*^	17/31 (55%) oral fluid	25/31 (81%)	15/31 (48%)	n.t.	n.t.	n.t.	Schommer et al. (5)
Adult^**^	0/6 (0%) saliva swabs	6/6 (100%)	n.t.	0/0 (0%)	n.t.	0/0 (0%)	Clark et al. (6)
Juvenile (3–5 weeks old)^**^	1/4 (20%)	3/4 (75%)	n.t.	2/4 (50%)	n.t.	0/4 (0%)	
10-day-old AaMP^***^	10/10 (100%) oral swabs	n.t.	n.t.	6/10 (60%)	7/10 (70%)	7/10 (70%)	Morozov et al. (8)

^*^Domestic and miniature, wild-type and genetically modified pigs. ^**^CD55 transgenic large white pigs. ^***^Aachen minipigs.

Recent in-depth investigations into the best way how, where, and when to screen for PCMV/PTV in donor pigs for xenotransplantation have shown that the most effective approach combines PCR testing with immunological assays (9). PCR testing of various pig samples, including oral fluids, is effective only in recently infected pigs and not in those where the virus has entered latency. Immunological testing, e.g., detection of specific antibodies against the virus, is the most effective method of identifying viral infections in adult animals when the virus is in its latent stage. In young animals, however, immunological testing may yield false-positive results due to the transfer of maternal antiviral antibodies through colostrum from an infected sow to her piglets. In such cases, PCR testing should be complemented by monitoring the time course of antiviral antibody titers. If the antibody titer declines to zero and the PCR results remain negative, the animal can be considered uninfected.

## Discussion

The study by Schommer et al. (5) makes a significant contribution to the development of efficient detection strategies for PCMV/PRV, particularly through the use of non-invasive samples from pigs. Oral fluids, which are easier to collect than oral or saliva swabs, would be an ideal sample for these purposes. I agree that herd monitoring using oral fluids can be convenient, such as utilizing a single rope to collect samples from multiple animals. However, the results indicate that this strategy is insufficient, particularly for adult pigs. While this method may be suitable for very young piglets, capturing them and collecting oral swabs would be a more effective approach to obtaining individual results. In all other cases, this method has to be combined with immunological testing. Especially in adult animals, where the virus is typically in its latent phase, screening with oral fluids or swabs will consistently yield false-negative results, as demonstrated in previous studies (6, 8). Therefore, oral fluid or swab testing should always be complemented by immunological testing to ensure accurate detection and identification of all infected animals.

Accurate testing and the correct selection of sample types for PCMV/PRV detection are crucial for xenotransplantation, and this screening should be conducted prior to xenotransplantation. Key steps to achieving this goal include eliminating the virus, establishing a virus-free herd, and maintaining strict herd isolation to prevent reintroduction. Since approximately 100% of German slaughterhouse pigs are infected with PCMV/PRV (10), the selection of animals for xenotransplantation requires specialized pathogen elimination programs (11). Accurate testing and the correct selection of sample types are crucial not only for xenotransplantation, where viruses can cause zoonoses or xenozoonoses in the transplanted non-human primate or human patient, but also for current clinical pig veterinary practices. This is true not only for PCMV/PRV, which, like other porcine viruses such as the porcine circovirus 3 and the porcine respiratory coronavirus, has only a limited clinical impact, but also for more pathogenic porcine viruses. The choice should be made based on the objective of sampling and testing and depending on the risk, which can be taken or assumed. In both contexts, xenotransplantation and veterinary practices, choosing the wrong sample type can lead to incorrect decisions with significant consequences.
